# Isolated Renal Echinococcosis with Hydatiduria in a Girl

**Published:** 2013-07-15

**Authors:** Vinod Priyadarshi, Jitendra Pratap Singh, Nipun kumar Awasthi, Shankar Prasad Hazra, Mukesh Kumar Vijay, Dilip Kumar Pal

**Affiliations:** Department of Urology, IPGMER and SSKM Hospital, Kolkata-20, India.

**Dear Sir,**

Hydatid disease is a common occurrence in the Indian subcontinent, however involvement of kidneys constitutes only about 2-4% of cases.[1] Isolated renal involvement without involvement of liver or lung is even more rare. Associated hydatiduria is seen in only 10-20% of all cases of renal hydatidosis and involvement of ureter is rarely reported.[2] Though, due to slow evolvement and asymptomatic nature, hydatid disease is rarely seen in children, one such isolated hydatid disease of right kidney and ipsilateral ureter is reported here in an adolescent girl who presented with recurrent renal colic and gross hydatiduria.

A fifteen year old girl referred to our institute for complaints of colicky pain in right lumbar region and dysuria with passage of small pale ruptured-grape like structures in urine for the last 6 months. Her general physical examinations and routine investigations were in normal limits. Ultrasonography of abdomen was suggestive of multiloculated cystic lesion, with typical spoke-wheel appearance, in the mid pole of right kidney and ELISA test was positive for echinococcus antibodies. CT scan obtained which revealed 11.5 cm X 11.0 cm multiseptated cystic lesion in mid and lower pole of right kidney and upper ureter with typical “cyst within the cyst” appearance (Fig. 1), without any contrast excretion from right kidney. With four weeks of albendazole therapy, surgical exploration done by flank extraperitoneal approach and right nephroureterectomy done as almost entire kidney was converted into a bag of cysts with multiple cysts filled up in the upper ureter as well (Fig. 2). Postoperatively, patient was put on albendazole (10 mg/kg daily) for eight weeks. Histopathological examination was consistent with hydatid disease. Follow-up ultrasound abdomen at 6 months showed no recurrence.

**Figure:**
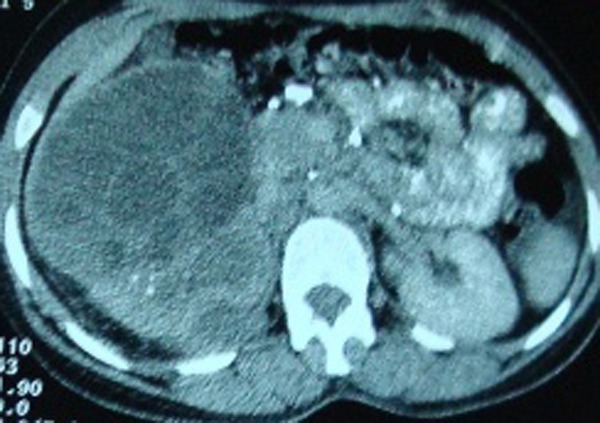
Figure 1:CT scan showing multiseptated cystic lesion in mid and lower pole of right kidney with cyst within the cyst appearance

**Figure:**
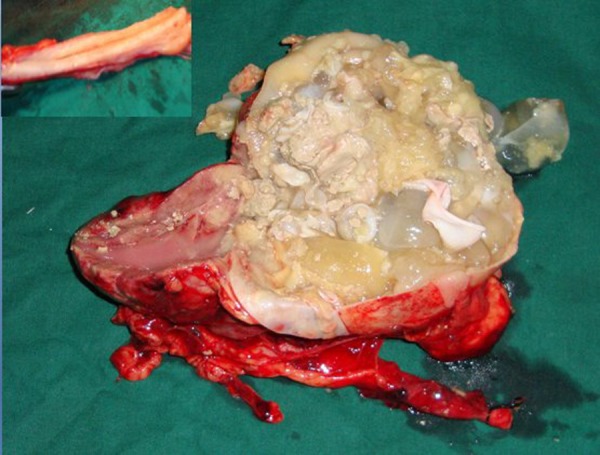
Figure 2:Bivalved specimen of hydatid cyst of right kidney and ureter (inset).

Renal hydatid cysts primarily develop when the larvae, which are not filtered in the liver, pass through the pulmonary circulation and travel to the kidney.[1] Presenting symptoms depend on the kind of cyst. Closed cyst that contains all 3 layers, pericyst, ectocyst, and endocyst, usually remain asymptomatic unless large enough to stretch renal capsule. Once the pericyst is lost, exposed cyst expands rapidly and presents as chronic dull flank pain. In communicating cysts, all the three layers of the cyst rupture with direct communication into the collecting system, causing hydatiduria and acute colic or obstruction but it is seen in only 10-20% of renal hydatidosis and is usually microscopic in nature.[1, 2]

There is no pathognomonic serological test for hydatid disease. Sonography is a sensitive method for the detection of membranes, septa, and hydatid sand within the cyst. Gharbi et al classified them into five types.[3] Present case, as having multiseptated daughter cysts, is a Type III cyst. Contrary to this, the most common variety reported in different paediatric series are Type I cysts, that have appearance of simple renal cysts but with double-contoured thick wall and hydatid sand as “falling snowflakes” [2, 3]. The CT scan has a higher accuracy and sensitivity to demonstrate the cyst wall and the daughter cysts.

Medical management with oral albendazole alone, is mostly unreliable and radiological intervention in the form of Percutaneous Aspiration Injection Re-aspiration followed by Percutaneous drainage (PAIR-PD) has limited success in only 70% of cases of unilocular cysts with vested fear of spillage and anaphylactic shock.[1, 4] Surgery remains the mainstay of treatment and a variety of surgical procedures are tailored to suit individual cases. Kidney sparing surgery i.e. marsupialization, closed total cystectomy, partial pericystectomy, or capitonnage with or without omentoplasty are possible in most (75%) cases.[1, 5] In other 25% cases, where the kidney is almost destroyed, nephrectomy is the only option.[1,2] In recent years, laparoscopic approach has also been used, but, the major problem with large renal hydatid cysts is the spillage of contents during dissection and the extent of the incision for the delivery of specimen.[1] Pre and postoperative one-month courses of albendazole should be considered in order to sterilize the cyst and to decrease the tension in the cyst wall that reduces the risk of spillage during surgery.[2] Though treatment is mostly surgical, it must be individualized.

## Footnotes

**Source of Support:** Nil

**Conflict of Interest:** None declared

